# Correction to: Downregulation of miRNAs during delayed wound healing in diabetes: role of Dicer

**DOI:** 10.1186/s10020-021-00348-6

**Published:** 2021-08-12

**Authors:** Sushant Bhattacharya, Rangoli Aggarwal, Vijay Pal Singh, Srinivasan Ramachandran, Malabika Datta

**Affiliations:** 1 Council of Scientific and Industrial Research (CSIR), Institute of Genomics and Integrative Biology, Mall Road, Delhi, 110007 India; 2 Academy of Scientific and Innovative Research (AcSIR), Kamala Nehru Nagar, Ghaziabad, 201002 India

## Correction to: Mol Med (2015) 21:847–860 10.2119/molmed.2014.00186

Following publication of the original article (Bhattacharya et al. 2015), the authors informed us that due to a human error, the Academy of Scientific and Innovative Research (AcSIR) has been missed to be added to this paper.

The author affiliation has been updated in this erratum.

## References

[CR1] Bhattacharya S, Aggarwal R, Singh VP, Ramachandran S, Datta M (2015). Downregulation of miRNAs during delayed wound healing in diabetes: role of Dicer. Mol Med.

